# Snake venom defensins: Defining the structural and functional characteristics of the toxin family

**DOI:** 10.1016/j.yjsbx.2025.100129

**Published:** 2025-05-27

**Authors:** David Melendez-Martinez, Adriana Morales-Martinez, Iliana Vanessa Almanza-Campos, Francisco Sierra-Valdez, Miguel Borja, Alejandro Carbajal-Saucedo, Christopher L. Parkinson, Jorge Benavides

**Affiliations:** a Tecnologico de Monterrey, The Institute for Obesity Research, Ave. Eugenio Garza Sada 2501 Sur, Monterrey, N.L., 64700, Mexico; b Tecnologico de Monterrey, Escuela de Ingeniería y Ciencias, Ave. Eugenio Garza Sada 2501 Sur, Monterrey, N.L., 64700, Mexico; c Centro de Investigación en Biotecnología, Universidad Autónoma del Estado de Morelos, Ave. Universidad 1001, C.P. 62209 Cuernavaca, Morelos, Mexico; d Universidad Autónoma de Nuevo León, Facultad de Ciencias Biológicas, Laboratorio de Herpetología, San Nicolás de los Garza, Nuevo León C.P. 66450, Mexico; e Facultad de Ciencias Biológicas, Universidad Juárez del Estado de Durango, Avenida Universidad s/n. Fracc. Filadelfia, C.P. 35010 Gómez Palacio, Durango, Mexico; f Department of Biological Sciences, Clemson University, Clemson, SC 29634, USA

**Keywords:** *Crotalus*, Crotamine-like peptides, K_v_ 1.3 channel, Myotoxin, Snake venom defensins

## Abstract

Snake venom defensins are a toxin family found in rattlesnake venoms (*Crotalus*) which are comprised of crotamine-like peptides and myotoxins. Their tertiary structure resembles the β-defensin family structure. Toxins from this family, such as crotamine (*C. durissus terrificus*) and myotoxin a (*C. viridis viridis*), have been described to generate paralysis through K_v_ 1.3 channel blockade, using three functional basic-hydrophobic dyads (Y-K, R-W, and R-W). However, the structural and functional properties of other snake venom defensins are scarcely described. For that reason, we evaluated the structural–functional characteristics of the rattlesnake venom defensins on the K_v_ 1.3 channel through *in silico* analysis. 38 snake venom defensins were found to be peptides from 41 to 48 residues with a highly conserved sequence. The three-dimensional structures had great similitude (RMSD, <1.1 Å). Moreover, molecular dynamics simulations showed that the structures were stable (0.445 ± 0.23 nm). It was found that the snake venom defensins contain two or three basic-hydrophobic dyads, the first one is present in the N-terminal region of the defensin comprised by YK. The dyads two and three are contiguous, forming a motif in the γ-core, of which there are seven phenotypes: RWKW, RWRW, PWRR, PWKR, RWKR, RLGW, and GWRR. These dyads played a key role in the interaction of the defensins with the pore residues of the K_v_1.3 channel. These results demonstrated that snake venom defensins have common structural and functional properties, interacting with the K_v_ 1.3 channel through the basic-hydrophobic dyads.

## Highlights

• Snake venom defensin 3D structure is highly similar (RMSD, <1.1 Å).
• Basic-hydrophobic dyads lead the venom defensin interaction with the K_v_ 1.3 channel.
• Exist 3 basic-hydrophobic dyads in the defensins, 2 are contiguous forming a motif.
• The basic-hydrophobic motif is represented by seven phenotypes.

## Graphical abstract

**Figure:**
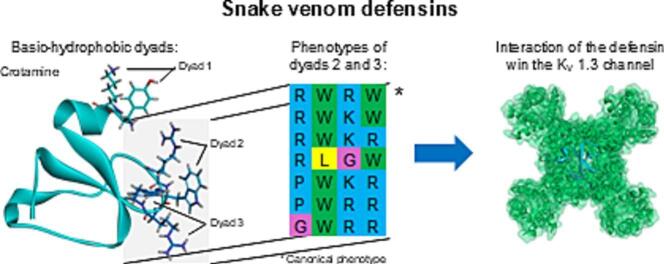

## Background

Crotamine-like peptides and myotoxins are peptides found in rattlesnake (*Crotalus* spp.) venoms (Bober et al., 1988). Both names refer to a single toxin family of peptides resembling the three-dimensional structure of the human β-defensin family (Coronado et al., 2013). Usually, the term crotamine-like peptide describes toxins isolated from South American rattlesnake venoms, whereas myotoxin refers to toxins from North American rattlesnake venoms. Nevertheless, the term “myotoxin” is also used to refer to toxins from any venomous animal that affect muscle activity. This is, independent from the mechanism involved or the toxin structure and includes, the toxins mentioned above plus cardiotoxins, and myotoxic phospholipases A_2_ (Gopalan et al., 2022, Lomonte et al., 2003, Porta et al., 2021). Therefore, we will use the name snake venom defensins to refer to crotamine-like peptides and myotoxins as a single family. Currently, snake venom defensins are defined as all the toxins present in *Crotalus* spp. venoms that resemble the three-dimensional structure of the human β-defensins, containing a conserved γ-core, 42–45 residue length, and six Cys forming three disulfide bridges (Porta et al., 2021).

Approximately 27 snake venom defensins have been isolated from *C. adamanteus*, *C. durissus cumanensis*, *C. d. ruruima*, *C. d. terrificus*, *C. oreganus concolor*, *C. o. helleri*, and *Crotalus viridis viridis* venoms (Porta et al., 2021). Moreover, snake venom defensin peptides have been identified in *C. basiliscus*, *C. catalinensis*, *C. culminatus*, *C. cerastes*, *C. d. cascabella*, *C. horridus*, *C. lepidus klauberi*, *C. mitchelli pyrrhus*, *C. molossus nigrescens*, *C. ornatus*, *C. ruber lucasensis*, *C. r. ruber*, *C. tigris*, *C. totonacus*, and *C. tzabcan* venoms through antibody recognition (ELISA and Western blot) (Bober et al., 1988, Borja et al., 2018, Ponce-López et al., 2021) or by proteomics, transcriptomics and genomics (Boldrini-França et al., 2010, Colis-Torres et al., 2022, Durban et al., 2017, Hofmann et al., 2018, Margres et al., 2021, Rokyta et al., 2012, Saviola et al., 2015, Schield et al., 2019). Demonstrating that the defensin family is present in the *Crotalus* genus and is not restricted to species with neurotoxic venoms.

Snake venom defensins have been identified as potent myotoxins that induce paralysis during envenomation (Oguiura et al., 2005), blocking the voltage-gated potassium (K_v_) channels 1.1, 1.2, and 1.3 (Peigneur et al., 2012). Particularly, the K_v_1.3 channels are located at the cell plasma membrane and control the cell K^+^ efflux. In excitable cells, they control the resting and action potentials, whereas in non-excitable cells regulate cell volume and proliferation (Gubič et al., 2021). For that reason, these channels are proposed for drug discovery to treat several health problems, including autoimmune diseases, chronic inflammation, obesity, type II diabetes, and cancer (Beeton et al., 2006, Comes et al., 2013, Pérez-Verdaguer et al., 2016). Conversely, crotamine from *C. d. terrificus* have demonstrated beneficial properties such as antimicrobial (Oguiura et al., 2011), antinociceptive (Mancin et al., 1998), antitumoral (Campeiro et al., 2018, Oyadomari et al., 2023), drug carrier through mammalian cell membranes (Nascimento et al., 2007), insulin secretion (Toyama et al., 2005), and anti-obesogenic (Campeiro et al., 2018, Marinovic et al., 2018, Melendez-Martinez et al., 2024). Crotamine, like other K_v_ channel toxins present in anemone and scorpion venoms, interact with the K_v_1.3 channel mainly through a residue arrangement called basic-hydrophobic dyads, which consist of a basic and a hydrophobic residue that can be either contiguous or near in the three-dimensional configuration. Crotamine is characterized by three basic-hydrophobic dyads consisting of Y1-K2, R31-W32, and R33-W34 (Melendez-Martinez et al., 2024, Peigneur et al., 2012). Among these, the R31-W32 dyad is the most relevant for the interaction of this toxin to interact with the K_v_1.3 channel (Melendez-Martinez et al., 2024).

However, the structural and functional properties of snake venom defensins other than crotamine are scarcely described. This gap needs further research to explore its molecular mechanisms and potential bioactivities, which could expand the understanding of their biological roles like envenomation and therapeutic applications such as antimicrobial, antinociceptive, antitumoral, drug carrier, insulin secretion, and anti-obesogenic. Therefore, we evaluated the structural–functional characteristics of 38 snake venom defensins using primary, secondary, and tertiary structural analysis, molecular dynamics simulations, and protein–protein docking with the K_v_1.3 channel. This structural–functional characterization will help to understand the role of snake venom defensins in producing neurotoxic symptoms during envenomation. Moreover, this will help to develop novel snake venom defensin-based drugs for biomedical applications such as antimicrobial, antinociceptive, antitumoral, drug carrier, type 2 diabetes, and obesity.

## Results

### Snake venom defensin sequences set

We obtained 38 snake venom defensin sequences (Table 1); of those, 28 were obtained from public databases, including Protein Data Bank, Uniprot, and NCBI. The other 10 sequences were obtained from rattlesnake genomes. These sequences are distributed among seven rattlesnake species, as follows: *C. durissus* (11 sequences), *C. adamanteus* (3 sequences), *C. basiliscus* (2 sequences), *C. oreganus* (9 sequences), *C. molossus* (6 sequences), *C. ornatus* (2 sequences), and *C. viridis* (5 sequences).

**Table 1:** Snake venom defensin sequences.

**Species**	**Name**	**Abbreviation**	**Accesion**	**MW (kDa)**	**pI**	**RMSD**
*C. adamanteus*	Myotoxin 1	Myo_adam_1	1801364A^a^	4.860	9.12	0.282 ± 0.035
	Myotoxin 2	Myo_adam_2	AEJ31978.1^a^	4.816	9.33	0.241 ± 0.034
	Myotoxin 3	Myo_adam_3	P24330^b^	5.516	9.33	0.457 ± 0.036
*C. basiliscus*	Myotoxin-1	Myo_basi_1	Gene^c^	7.332	8.71	1.180 ± 0.040
	Myotoxin-2	Myo_basi_2	Gene^c^	5.560	9.09	0.394 ± 0.034
*C. durissus cumanensis*	Crotamine-IV-2	Ctm_ducu_1	P86193^b^	4.907	9.28	0.337 ± 0.036
	Crotamine-IV-3	Ctm_ducu_2	P86194^b^	4.958	9.28	0.616 ± 0.050
*C. d. ruruima*	Crotamine Ile-19	Ctm_duru_1	P63327^b^	4.890	9.09	0.519 ± 0.039
*C. d. terrificus*	Crotamine	Ctm_dute_1	AAF34911.1^a^	5.018	9.26	0.310 ± 0.045
	CRO1	Ctm_dute_2	O57540^b^	5.018	9.26	0.530 ± 0.043
	CRO3	Ctm_dute_3	O73799^b^	4.855	9.37	0.411 ± 0.079
	Myotoxin-1	Ctm_dute_4	P24331^b^	4.814	9.12	0.329 ± 0.036
	Myotoxin-2	Ctm_dute_5	P24332^b^	4.808	9.49	0.499 ± 0.033
	Myotoxin-3	Ctm_dute_6	P24333^b^	4.742	9.33	0.316 ± 0.037
	Myotoxin-4	Ctm_dute_7	P24334^b^	5.117	9.44	0.357 ± 0.040
	Crotamine	Ctm_dute_8	Q9PWF3^b^	4.726*	9.54*	0.395 ± 0.054
*C. molossus molossus*	Myotoxin-1	Myo_momo_1	Gene^c^	5.464	9.28	0.291 ± 0.021
*C. m. nigrescens*	CMN MYO-1	Myo_moni_1	Gene^c^	5.450	9.28	0.305 ± 0.036
	CMN MYO-3	Myo_moni_2	Gene^c^	5.464	9.28	0.517 ± 0.054
	CMN MYO-5	Myo_moni_3	Gene^c^	5.560	9.09	0.290 ± 0.051
	CMN MYO-6	Myo_moni_4	Gene^c^	5.518	9.33	0.587 ± 0.115
	CMN MYO-7	Myo_moni_5	Gene^c^	5.419	9.5	0.509 ± 0.091
*C. oreganus concolor*	Myotoxin-1	Myo_orco_1	P12028^b^	5.061	9.09	0.325 ± 0.025
	Myotoxin-2	Myo_orco_2	P12029^b^	5.034	9.24	0.378 ± 0.048
*C. o. helleri*	Crotamine 1	Ctm_orhe_1	AEU60009.1^a^	5.545	9.24	0.275 ± 0.019
	Crotamine 2	Ctm_orhe_2	AEU60010.1^a^	7.347	9.67	1.482 ± 0.051
	Crotamine 3	Ctm_orhe_3	AEU60011.1^a^	5.464	9.28	0.365 ± 0.069
	Crotamine 4	Ctm_orhe_4	AEU60012.1^a^	5.488	9.28	0.281 ± 0.027
	Crotamine 5	Ctm_orhe_5	AEU60013.1^a^	4.961	9.44	0.505 ± 0.058
	Crotamine 6	Ctm_orhe_6	AEU60014.1^a^	4.990	9.23	0.387 ± 0.085
	Crotamine 7	Ctm_orhe_7	AEU60015.1^a^	5.018	9.39	0.481 ± 0.050
*C. ornatus*	Myotoxin-1	Myo_orna_1	Gene^c^	5.464	9.28	0.483 ± 0.048
	Myotoxin-8	Myo_orna_2	Gene^c^	5.488	9.28	0.307 ± 0.016
*C. viridis viridis*	Myotoxin-A	Myo_vivi_1	P01476^b^	4.828	9.06	0.309 ± 0.035
	Myotoxin-2	Myo_vivi_2	P63175^b^	5.246	9.09	0.444 ± 0.044
	Myotoxin-3	Myo_vivi_3	P63176 b	5.174	9.28	0.414 ± 0.031
	Myotoxin a	Myo_vivi_4	JC5324^a^	4.947	9.23	0.416 ± 0.061
	Myotoxin 2	Myo_vivi_5	1702207A^a^	5.246	9.09	0.273 ± 0.019

Notes:

* Results obtained Kerkis *et al*. (Kerkis et al., 2014).

a Accesion from GeneBank.

b Accesion from Uniprot.

c Putative aminoacidic sequences obtained from genes (unpublished data).

### Primary structure diversity of snake venom defensins

**Fig. 1:**
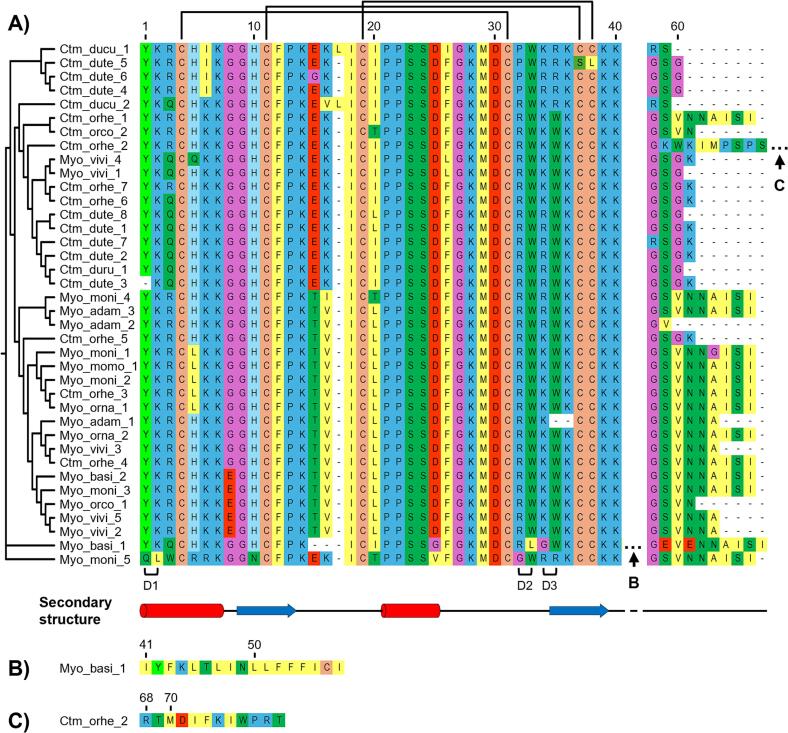
Phylogenetic tree and multiple sequence alignment of snake venom defensins. A) Phylogenetic tree and multiple sequence alignment of the snake venom defensins, Myo_moni_5 (B) and Ctm_orhe_2 (C) demonstrated to have unique sequence fragments in the C-terminal of the sequence. The secondary structure of the snake venom defensins was obtained from Crotamine (Ctm_dute_8) (Fadel et al., 2005), α-helices are denoted as red boxes and β-strands as blue arrows. B) Unique sequence fragment of Myo_moni_5 sequence, from residue 40 to 56. C) Unique sequence fragment of Ctm_orhe_2 sequence, from residue 68 to 79. The phylogeny was built using the mature peptide of the snake venom defensins by the UPGMA method (Bootstrap replicates: 1,000). The multiple sequences alignment was made using MUSCLE (Multiple sequence comparison by Log-expectation) algorithm. In the lower margin of the multiple sequence alignment are identified the three basic-hydrophobic dyads (marked as D1, D2, and D3) described for crotamine: D1, Y1-K2; D2, R32-W33; D3, R34-W35. (For interpretation of the references to colour in this figure legend, the reader is referred to the web version of this article.)

Similar to other members of the group, most of the snake venom β-defensins show the conserved six Cys residues implicated in the formation of three disulfide bonds (Fig. 1). However, two noticeable exceptions were Myo_basi_1 from *C. basiliscus* with a seventh Cys residue at position 56, which could provoke a strikingly new Cys-Cys rearrangement. In Ctm_dute_5 from *C. d. terrificus*, the conserved fifth and sixth cysteines have been replaced by Ser and Leu residues at position 37 and 38, respectively, which provoked the loss of the second and third disulfide bond bonds.

Rattlesnake venom defensins demonstrated an average length of 45 residues (Fig. 1). Most of the sequences ranged from 41 to 48 residues, and two sequences, Myo_basi_1 from *C. basiliscus* (67 residues) and Ctm_orhe_2 from *C. o. helleri* (79 residues) showed unusually long lengths. The *C. o. helleri* toxin showed a 17 amino acids insertion at position 41 within the conserved basic motif (KKG/R), while Myo_basi_1 possesses a 12 residue extension at the C-terminal region (Fig. 1**B and C**).The Myo_moni_5 from *C. m. nigrescens* also has noticeable features in comparison to the other snake venom defensins (Fig. 1**A**), having major differences in the basic-hydrophobic dyad 1, where the conserved Tyr has changed by Gln, and Lys, at position 2, has changed by Leu. Also, an essential modification of the first position of dyad 2, residue 32, has resulted in the mutation of the common Arg residues to Gly. At the third dyad, Arg residue at position 35 instead of Trp, shows to be a shared characteristic exclusively present in Myo_moni_5 and defensins from *C. d. terrificus*.

The three basic-hydrophobic dyads show different phenotypes among snake venom defensins. Two phenotypes, Y-K (37 sequences out of 38) and Q-L (1/38) were observed for the first dyad (residues 1 and 2). Four phenotypes were found for the second dyad (positions 32–33): RW (32/38), PW (4/38), RL (1/38), and GW (1/38) and six phenotypes were found for the third dyad (position 34–35): KW (21/38), RW (9/38), RR (4/38), KR (2/38), K- (1/38), and GW (1/38). These results suggest that every dyad plays different roles in recognition against the molecular target. In this context, the first dyad is of major importance due to the high degree of conservation among all rattlesnake defensins. In contrast, the third dyad, showing the highest variability in the primary sequence, could play a complementary role.

### Three-dimensional structure diversity of snake venom defensins

**Fig. 2:**
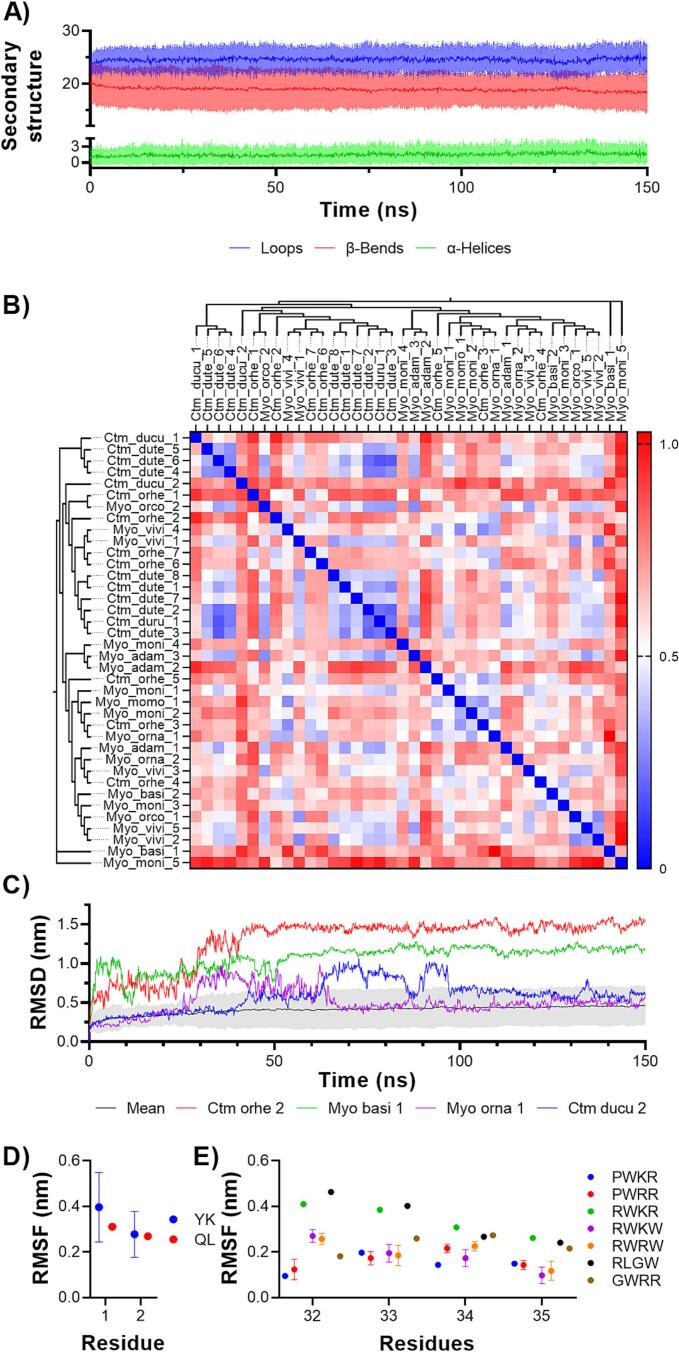
**Secondary and tertiary structure similarity and stability of snake venom defensins. A)** Average abundance of secondary structures in snake venom defensins during 150 ns of molecular dynamics simulation. The mean and the standard deviation values are represented by solid lanes and shaded areas, respectively. **B)** Structural comparison of snake venom defensins through RMSD. **C)** Structural stability of the snake venom defensins, the average stability was compared with the most unstable structures (Ctm_orhe_2, Myo_basi_1, Myo_orna_1, and Ctm_ducu_2) to demonstrate the structural diversity of the toxin family. The effect of the dyad phenotype flexibility of the dyads **D)** 1, **E)** 2, and 3 was observed in panels G and H through RMSF.

A comparative analysis of the three-dimensional structures of snake venom defensins revealed that key physicochemical properties, such as molecular weight and isoelectric point (pI), are highly conserved across this family. The molecular mass of the toxin family was 5.27 ± 0.56 kDa (ranging from 4.74 to 7.35 kDa), and pI was 9.25 ± 0.16 (ranging from 8.71 to 9.67) (Table 1). All the three-dimensional structures exhibited a conserved structure consistent with canonical Crotamine (Ctm_dute_8) from *C. d. terrificus*. Molecular dynamics simulations evidenced that all snake venom defensins are comprised mostly of loops and β-bends, followed by α-helices (Fig. 2**A**).

The temporal evolution of the three-dimensional structure of the snake venom defensins was monitored using C-α RMSD analysis. This approach provides quantitative insights into structural stability and conformational changes throughout the simulation; this parameter remained < 1.1 Å (0.612 ± 0.160 Å) in all the comparisons made (Fig. 2**B**), suggesting a high similarity among all the snake venom defensins. The snake venom defensins from the *C. durissus* complex (0.465 ± 0.182 Å), including *Ctm_dute_1-8* and *Ctm_duru_1*, exhibited a high structural identity, with RMSD values below 0.50 Å. In contrast, two defensins from this complex, *Ctm_ducu_1* (0.658 ± 0.087 Å) and *Ctm_ducu_2* (0.722 ± 0.043 Å) from *C. d. cumanensis* venom displayed a slight increment of RMSD values. This suggests that the additional Leu residue at position 17 (Fig. 1**A**) may have a minimal influence on their three-dimensional structure. The molecular dynamics analysis on *C. durissus* complex defensins demonstrated that the extra Leu17 incremented the flexibility only in the *Ctm_ducu_2* γ-core (residues 20–34), disturbing its stability **(Supplementary figure 1).** In contrast, the black-tailed rattlesnake species complex (*C. m. molossus*, *C. m. nigrescens*, and *C. ornatus*) demonstrated to share structural similitude in their snake venom defensin three-dimensional structures (0.538 ± 0.106 Å). On the other hand, we observed that Ctm_ducu_2 (0.762 ± 0.77 Å), Ctm_orhe_1 (0.744 ± 0.140 Å), Myo_adam_2 (0.736 ± 0.136 Å), Myo_basi_1 (0.723 ± 0.107 Å), and Myo_moni_5 (0.830 ± 0.120 Å) were the defensins with higher C-α RMSD fluctuations, suggesting that were the most different structures among snake venom defensins.

**Fig. 3:**
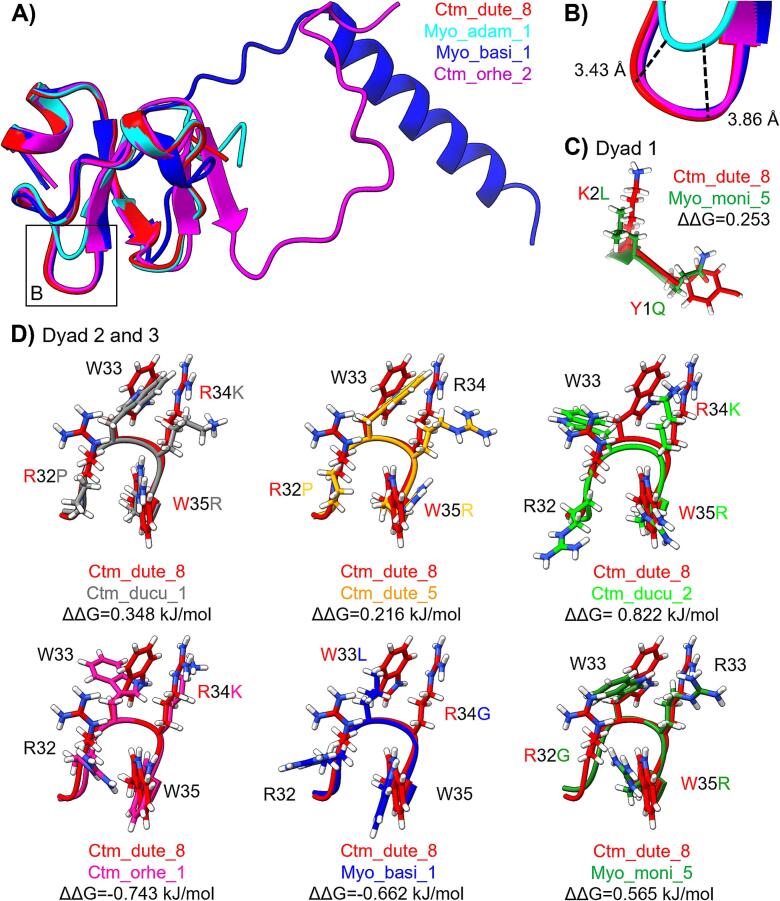
**Three-dimensional stability of the basic-hydrophobic dyads in snake venom defensins. A)** Structural alignment of the most representative variations in snake venom defensins. The average snake venom defensin structure is represented by Ctm_dute_8 (red), structural modifications were observed in Myo_basi_1 (blue) and Ctm_orhe_2 (magenta), and Myo_adam_1 (cyan). **B)** Zoom of the γ-core shortening presented in Myo_adam_1. In panels **C** and **D**, the effect of the mutations on basic-hydrophobic dyads of the snake venom defensins stability was measured using the free energy change (ΔΔG, kJ/mol) as an indicator, crotamine from *C. d. terrificus* (Ctm_dute_8) was used as a canonical structure to compare with the other snake venom defensin structures. **C)** The effect of the mutations on the basic-hydrophobic dyad 1 on Myo_moni_5 (green) residues, Y1K and K2L. **D)** On the basic-hydrophobic dyads 2 and 3 (from residue 32 to 35) seven phenotypes were observed RWRW (native, Ctm_dute_8 in red), PWKR (Ctm_ducu_1 in grey), PWRR (Ctm_dute_5 in orange), RWKR (Ctm_ducu_2 in lime), RWKW (Ctm_orhe_1 in pink), RLGW (Myo_basi_1 in blue), and GWRR (Myo_moni_5 in green). (For interpretation of the references to colour in this figure legend, the reader is referred to the web version of this article.)

The geometric optimization of the snake venom defensins were assayed through molecular dynamics simulations RMSD during 150 ns. From the last 50 ns of simulations, mostly all the defensins were found to remain stable structurally (0.445 ± 0.23 nm, **Supplementary Fig. 2**), but Ctm_ducu_2 (100 ns) and Myo_orna_1 (70 ns) required more simulation time to reach their stability (Fig. 2**C**). Moreover, Myo_basi_1 and Ctm_orhe_2 structures reached stability with higher RMSD values, 1.482 ± 0.051 and 1.180 ± 0.040 respectively. These results relate to the C-terminal overextension in these defensins (Fig. 3**A**). Finally, Myo_adam_1 had a 3.43–3.86 Å smaller γ-core structure due to a lack of residues in positions 15 and 16 (Fig. 3**A and B**). Still, it did not affect its stability (Fig. 2**B** and **Supplementary Fig. 2A**).

To evaluate the effect of the mutations in the snake venom defensins, the change of free energy (ΔΔG, kcal/mol) was measured, using as control the canonical three-dimensional structure of Crotamine (Ctm_dute_8) from *C. d. terrificus*. Most mutations did not negatively affect the defensin stability (**Supplementary Fig. 3**). The mutations that proved to be destabilizing were observed in residues 1, 35, 37, 38, and 59. Nevertheless, the mutations of C37S and C38L did not show any effect on Ctm_dute_5 stability or Cα-residues fluctuation (**Supplementary Fig. 2**). Moreover, we evaluated the stability and flexibility of the basic-hydrophobic dyads phenotypes. In dyad 1 (Fig. 3**C**), the mutations observed in Myo_moni_5 (K2L and Y1Q) destabilized this area of the defensin (ΔΔG, 0.253 kcal/mol), but separately, K2L mutation resulted to be stabilizing (**Supplementary Fig. 3**). These mutations did not affect the flexibility of these residues significantly (Fig. 2**D**).

From now on, the phenotypes in dyads 2 and 3 will be presented as a single motif (Fig. 3**D**). The phenotypes RWKW and RLGW demonstrated greater structural stability, with free energy changes (ΔΔG) of −0.743 and −0.662 kcal/mol, respectively, compared to the canonical RWRW. All other analyzed phenotypes exhibited destabilizing effects. On an individual residue level, the mutations R32P, R32G, W33L, R34K, and W35R may contribute to increased stability (**Supplementary Fig. 3**). However, the mutation W35R showed a destabilizing effect, with a ΔΔG of 1.421 kcal/mol. This destabilization likely accounts for the negative impact observed in phenotypes PWKR, PWRR, RWKR, and GWRR, particularly affecting the structural integrity of dyads 2 and 3. These mutations on dyads 2 and 3 were demonstrated to modify the flexibility of these residues. Particularly, the phenotypes containing P32 (PWKR and PWRR) restricted the mobility of this β-turn. On the other hand, the phenotypes that only included one aromatic residue (RWKR, RLGW, and GWRR) had a higher flexibility (Fig. 2**E**).

### Snake venom defensins interaction with the K_v_1.3 channel

**Fig. 4:**
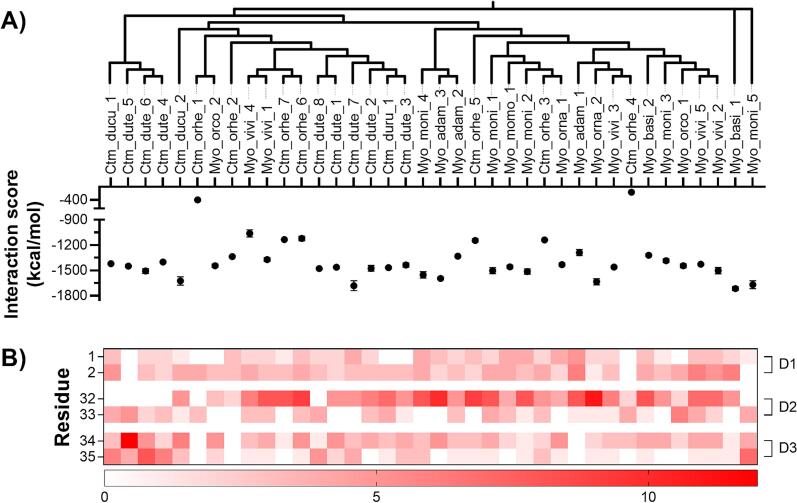
**Snake venom defensins interaction with the K_v_1.3 channel.** The resulting dockings were analyzed to obtain the **A)** Interaction score (ΔG, kcal/mol) of the snake venom defensin when interacting with the K_v_1.3 channel. The snake venom defensins were arranged according to the phylogeny made in Fig. 1. **B)** The heatmap illustrates the number of interactions generated by the basic-hydrophobic dyads (D1, Y1-K2; D2, R32-W33; D3, R34-W35) in the snake venom defensin- K_v_1.3 channel interaction.

The functional activity of the snake venom defensins was evaluated through protein–protein docking using as receptor the K_v_1.3 channel. The snake venom defensins affinity was determined through the interaction score (kcal/mol) calculated by ClusPro 2.0 (Kozakov et al., 2017). It was found that all the snake venom defensins were able to interact with the K_v_1.3 channel, ranging from −382.4 to −1,715.34 kcal/mol (Fig. 4**A**). The snake venom defensins that demonstrated the highest affinity to the K_v_1.3 channel were Myo_basi_1 (−1,715.34 ± 22.33 kcal/mol), Ctm_dute_7 (−1,681.42 ± 57.83 kcal/mol), and Myo_moni_5 (−1,669.34 ± 46.38 kcal/mol), whereas the defensins with lower affinity were Ctm_orhe_4 (−382.4 ± 0.94 kcal/mol) and Ctm_orhe_1 (−400.14 ± 1.98 kcal/mol).

In all snake venom defensins, the three basic-hydrophobic dyads (residues 1–2, 32–33, 34–35) interacted with the K_v_1.3 channel (Fig. 4**B, Supplementary Fig. 4**). The basic residues 2, 32, and 34 were the most relevant, as these generated most of the interactions, followed by the aromatic residues 1, 33, and 35. However, other basic residues of defensins such as R3, H5, K/R6, K14, and K40 (**Supplementary Fig. 4**) seem to show relevant interaction with this ion channel. In dyad 1, substituting the canonical basic-hydrophobic YK motif to QL in Myo_moni_5 diminished the number of interactions of this dyad with the K_v_1.3 channel. However, this loss was partially compensated by the interactions involving residues R5 and R6. In dyads 2 and 3, phenotypes that contained P32 (PWRR and PWKR) diminished the number of interactions generated by the residue in this position. In the case of phenotype RLGW in Myo_basi_1, the mutations W33L and R34G negatively affected the involvement of dyads 2 and 3, leading to a loss of interactions of the dyads. This demonstrates that an increased number of basic residues (R and K) in the dyad correspond to more interactions.

Analyzing the impact of the dyads 2 and 3 (residues 32–35) phenotype diversity in the interaction with the K_v_1.3 channel, we did not observe any statistical difference in the energies of interaction. Moreover, the number of dyads present in this motif did not restrict the interaction with this channel (Fig. 4**A**). Nevertheless, the extended C-terminal regions in some of the snake venom defensins demonstrated to play a role in maintaing the interaction with the K_v_1.3 channel (Ctm_orhe_2, Myo_basi_1, and Myo_moni_5, **Supplementary Fig. 4**). Finally, although the smaller γ-core domain observed in Myo_adam_1 (Fig. 3**B**) did not significantly alter its overall affinity for the K_v_1.3 channel (Fig. 4**A**), it resulted in the loss of interactions between the basic-hydrophobic dyad 3 and the channel (Fig. 4**B**).

**Fig. 5:**
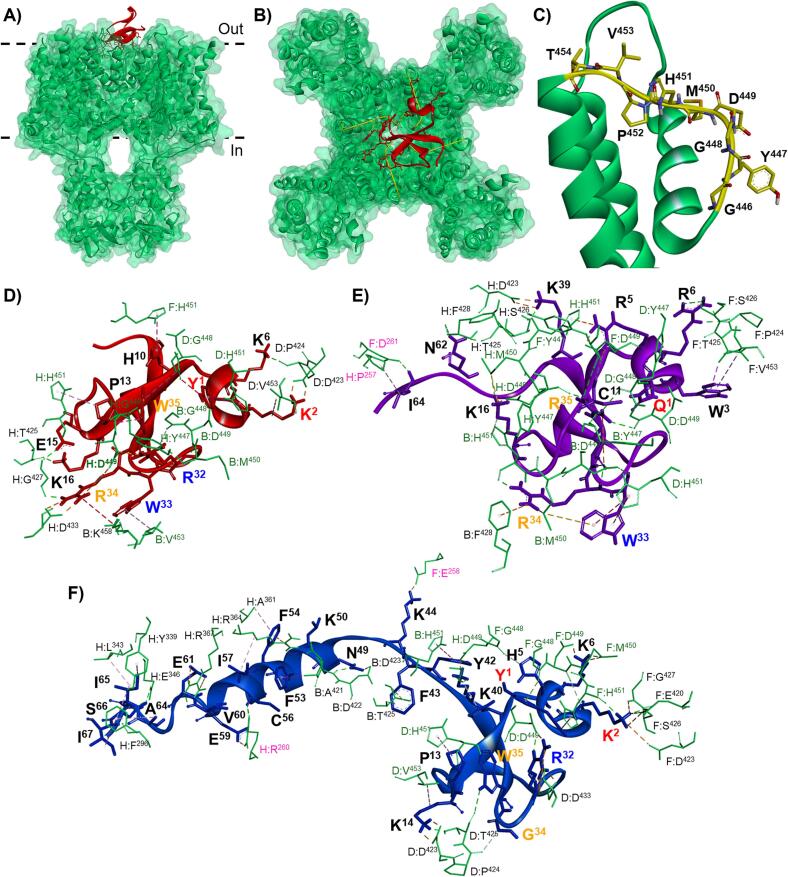
**Illustration of the interaction of snake venom defensins with K_v_1.3 channel.** Three-dimensional illustration of the canonical Crotamine (Ctm_dute_8) interaction from *C. d. terrificus*, from **A)** side view and **B)** top view. **C)** Visualization of the transmembrane helices S5 and S6 from K_v_1.3 channel ion-conducting pore, the pore residues G446-T454 (highlighted in yellow) from the ion channel were relevant for the interaction with the snake venom defensins. Visualization of the interactions generated by **D)** the canonical Crotamine (Ctm_dute_8), and two of the snake venom defensins with the highest affinity to the K_v_1.3 channel, **E)** Myo_moni_5 (−1,669.34 ± 46.38 kcal/mol) and **F)** Myo_basi_1 (−1,715.34 ± 22.33 kcal/mol). In panels **D**, **E**, and **F** the snake venom defensin residues relevant for the interaction are shown, residues from the basic-hydrophobic dyads 1, 2, and 3 are showed in red, blue, and orange, respectively. K_v_1.3 channel residues are represented in green, the residues that are part of the pore region are highlighted in green and the residues from the voltage-sensing domain are highlighted in pink. (For interpretation of the references to colour in this figure legend, the reader is referred to the web version of this article.)

The interaction of snake venom defensins were observed in the extracellular face of the K_v_1.3 channel (Fig. 5**A and B**). Mainly, the toxins interacted with the pore region of the ion channel (Fig. 5**B**), interacting with the residues 446-GYGDMHPVT (Fig. 5**C**) thought hydrophobic dyad 2 and 3 (**Supplementary Fig. 5**). Additional interactions were found with the residues 419-AEADDPTSGFS, which were also present in the extracellular face of the channel. Snake venom defensins were more likely to interact with the K_v_1.3 channel pore region through dyads 2 and 3 than with dyad 1 (Fig. 5**D-F**). Finally, the long C-terminal snake venom defensins, Myo_moni_5 (−1,669.34 ± 46.38 kcal/mol) and Myo_basi_1 (−1,715.34 ± 22.33 kcal/mol), were able to interact with the voltage-sensing domain residues P257, E258, R260, and D261 (Fig. 5**E and F**).

## Discussion

Snake venom defensins are a toxin family that resembles the structure of the β-defensins from several animals, including humans (Yount and Yeaman, 2006). Adding to this structural similarity to the β-defensins and the myotoxic activity (*e*.*g*., rigid paralysis) displayed during rattlesnake envenomation, it is reported that crotamine (Ctm_dute_8) has antimicrobial and antifungal activities (Hayashi et al., 2022, Yount and Yeaman, 2006). These similarities suggest that this toxin family underwent a neofunctionalization event from non-toxic β-defensins to snake venom defensins, adding myotoxicity as a novel property for predatory activities (Porta et al., 2021). Therefore, they may have a dual role in rattlesnakes: defensive against microorganisms and predators and offensive to immobilize the prey.

The analysis of the primary, secondary, and tertiary structure of the snake venom defensins demonstrated that this toxin family among the *Crotalus* genus is highly preserved (Fig. 1, Fig. 2, Fig. 3). Our results of the secondary structure analysis demonstrated that the snake venom defensins are comprised of loops, β-bends, and α-helices, which agrees with the previously reported structure for Crotamine (Ctm_dute_8) from *C. d. terrificus* using nuclear magnetic resonance spectroscopy (Fadel et al., 2005). The helix may appear in the N-terminal (residues 1–7), and the β-bends are present forming the strands in the antiparallel β-sheet (residues 9–13 and 35–39). Finally, the loops are conformed for the residues connecting the antiparallel β-sheets (residues 1–34) and in the C-terminal (from residue 40).

The three-dimensional structure is conserved among the snake venom defensin diversity tested here. Nevertheless, *C. d. cumanensis* snake venom defensins have an additional Leu in position 17, but this addition only has minimal modifications on *Ctm_ducu_2* stability and did not affected its interaction with the K_v_1.3 channel. The mutations observed in basic-hydrophobic dyads 2 and 3 (residues 32–35) generated seven phenotypes: RWRW, PWKR, PWRR, RWKR, RWKW, RLGW, and GWRR. Some of these mutations restricted the number and position of the functional dyads from the common RW or KW to a reversed dyad WK or WR (phenotypes PWKR, PWRR, RWKR, and GWRR). This reversed WK/R dyad was functional for the snake venom defensins that contain this feature when interacted with K_v_1.3 channel (−1,400.98 to −1,669.34 kcal/mol, Fig. 4**A**).

Previous studies denoted that crotamine (Ctm_dute_8) has three basic-hydrophobic dyads (Melendez-Martinez et al., 2024). Here, we demonstrated that snake venom defensins have two or three functional basic-hydrophobic dyads. The presence of the basic-hydrophobic dyads is a common feature in K_v_ channel toxins, as observed in other venomous animals such as anemones and arachnids. In the case of the anemone and arachnid K_v_ channel toxins, these toxins have only one basic hydrophobic dyad conformed by Tyr and Lys (Chandy et al., 2023, Gubič et al., 2021, Naseem et al., 2022, Peigneur et al., 2012, Sanches et al., 2023, Zhang et al., 2022). Moreover, as we previously denoted, the dyad present in these toxins can be formed by consecutive or non-consecutive residues (Melendez-Martinez et al., 2024). The presence of the two additional basic-hydrophobic dyads in snake venom defensins may be an evolutionary trait to increase the affinity of this toxin family to the K_v_ channel, as these toxins have higher affinity for this channel in comparison to the anemone and arachnid venom toxins (Khalili-Salmasi et al., 2024, Rashid and Kuyucak, 2012).

Snake venom defensins interacted with the K_v_1.3 channel, as described previously for Crotamine (Ctm_dute_8) (Peigneur et al., 2012). Interestingly, even when *C. d. terrificus* venom is considered one of the most lethal rattlesnake venoms (Calvete et al., 2010) due to the presence of crotamine; this toxin did not have the highest affinity to the K_v_1.3 channel (Fig. 3**A**). Two snake venom defensins with the highest affinity to the K_v_1.3 channel were obtained from the North American rattlesnakes, *C. basiliscus* (Myo_basi_1) and *C. m. nigrescens* (Myo_moni_5). Interestingly, both species possess snake venom defensins in juvenile specimens (Borja et al., 2018, Colis-Torres et al., 2022). However, both snake venom defensins were deduced from genes, and the confirmation of their presence in the venom is needed.

The interaction of the snake venom defensins with the K_v_1.3 channel and other possible receptors, such as glucagon-like peptide-1 receptor, dipeptidyl peptidase IV, and α-glucosidase is mainly mediated by the basic-hydrophobic dyads (Melendez-Martinez et al., 2024, Peigneur et al., 2012, Yount and Yeaman, 2006). Our results demonstrated that dyad 2 (residues 32–33) was the most relevant dyad to involved in the interaction with the K_v_1.3 channel, followed by dyad 3 (residues 34–35). This is contrary to what is described for crotamine (Ctm_dute_8) (Melendez-Martinez et al., 2024, Yount and Yeaman, 2006). Residues 34–35 were only relevant to snake venom defensins that contained the reversed functional dyad (residues 33–34). Nevertheless, the differences on the phenotypes among the snake venom defensins affected in number of interactions per defensin residue, not the interaction score. Finally, all the snake venom defensins could interact with the K_v_1.3 channel pore, particularly with Y447, G448, D449, and M450, which are relevant for K^+^ ion efflux (Selvakumar et al., 2022). This suggests that the mechanism of action for snake venom defensins to produce paralysis or the therapeutic effects is through the K_v_1.3 channel blockade.

## Conclusions

This work highlighted the common structural and functional features of the snake venom defensins toxin family, demonstrating that all the members of this toxin family are capable to interact with the K_v_1.3 channel pore residues responsible for K^+^ ion efflux (Y447, G448, D449, and M450). This interaction was led mostly by the basic-hydrophobic dyads, usually comprised by RW or KW. Furthermore, we observed a fully functional new dyad arrange, a reversed dyad WK/R in some of the snake venom defensins. This structural–functional molecular insights will help to understand the role of snake venom defensins in producing neurotoxic symptoms during envenomation. Moreover, this will help to develop novel snake venom defensin-based drugs for biomedical applications.

## Methods

### Sequence search and curation

The sequence search was conducted in several databases, including Protein Data Bank (https://www.rcsb.org/), UniProt (https://www.uniprot.org/), and NCBI (https://www.ncbi.nlm.nih.gov/). The search employed the keywords “Crotamine”, “Crotamine-like peptide” and “Myotoxin”. The obtained sequences were curated manually to retain those derived from the *Crotalus* genus and containing complete peptide sequences. The remaining sequences were downloaded in FASTA format and trimmed to conserve the mature peptide region.

The putative amino acid sequences of snake venom defensins for *C. basiliscus* (CBA07*)*, *C. m. molossus* (CMM06), *C. m. nigrescens* (CMN46), and *C. ornatus* (CO08) were inferred from genomes of representative individuals from each lineage (unpublished data).

### Multiple sequence alignment and phylogenetic analysis

Snake venom defensin sequences were aligned using MUSCLE (Multiple sequence comparison by Log-expectation) multiple sequence algorithm (Edgar, 2004). MUSCLE was performed using MEGA-X software version 10.1 (Kumar et al., 2018). The phylogenic tree was made using the UPGMA (Unweighted pair group method with arithmetic mean) algorithm, based on the Jones-Taylor-Thornton substitution model (Jones et al., 1992) and a bootstrap phylogeny test with 1,000 replicates, using MEGA-X software (Kumar et al., 2018). The resulting phylogenetic tree was visualized and edited using iTOL version 6 (Letunic and Bork, 2021).

### Three-dimensional structure prediction

Three-dimensional structure prediction of snake venom defensins was performed using the Robetta server (Kim et al., 2004), using Crotamine from *C. d. terrificus* (PDB ID: 4GV5) as a template. The protonation of the resulting snake venom defensins PDB models was made using APBS-PDB2PQR software (Jurrus et al., 2018).

The pairwise structure comparison of the snake venom defensins was made through the matchmaker tool in ChimeraX 1.8 (Meng et al., 2006). The molecular weight and pI were calculated using the Prot pi Peptide tool (https://www.protpi.ch/Calculator/PeptideTool). The effect of the mutations on the structure stability was evaluated through the change of free energy (ΔΔG, kJ/mol) using the MAESTROweb server (Laimer et al., 2016).

### Molecular dynamics simulations

The geometric optimization of snake venom defensins was conducted through molecular dynamics simulations. Simulation models were constructed with CHARMM-GUI (Qi et al., 2014), incorporating peptides in solution modeled with the TIP3 water, at physiological conditions (pH 7.4) and saline concentration of 0.15 M NaCl, establishing CHARMM36m as a force field.

Simulation stages run in GROMACs 2019.3 (Abraham et al., 2015, Lindahl et al., 2019, Páll et al., 2015). The first stage involved a 5000-step energy minimization to resolve steric clashes and stabilize the system. The second stage comprised two equilibrium phases: the first, conducted under an NVT ensemble (constant number of particles, volume, and temperature) for 125 picoseconds, and the second under an NPT ensemble (constant number of particles, pressure, and temperature) for 2 ns (Bekker et al., 1995, Berendsen et al., 1995, Darden et al., 1993). The final stage consisted of a 150-nanosecond production run, during which flexibility assessments and secondary structure analyses of the defensin molecules were performed.

### Protein-protein molecular docking

The K_v_1.3 channel was selected as receptor as these are described as the molecular target of Crotamine (Peigneur et al., 2012). The selected model (PDB ID: 7EJ1) was obtained from Protein Data Bank (https://www.rcsb.org/pdb/) (Berman, 2000). The model geometry and stereochemistry were evaluated through MolProbity (Williams et al., 2018), the missing residues were restored using the Robetta server (Kim et al., 2004), and the model protonation was made using APBS-PDB2PQR software (Jurrus et al., 2018). The snake venom defensin models previously constructed for structural analysis were used to perform molecular docking with the K _v_1.3 channel.

Protein-protein docking of snake venom defensins and the K_v_1.3 channel was carried out using the ClusPro 2.0 server (https://cluspro.bu.edu/home.php) (Desta et al., 2020, Kozakov et al., 2017). The molecular docking was directed to K_v_1.3 channel residues S426, Y447, G448, D449, M450, H451, P452, and T454 from chains B, D, F, and H, as these residues have demonstrated to be relevant for K_v_1.3 channel pore blocking (Peigneur et al., 2012, Sanches et al., 2023). The resulting docking poses and interaction scores (kcal/mol) for each snake venom defensin-K_v_1.3 channel docking were downloaded. The models were inspected visually to select the models where snake venom defensins interacted with the inhibitory site of the K_v_1.3 channel using BIOVIA Discovery Studio Visualizer 2021 software (Dassault Systèmes, San Diego, CA, USA). From these, the models with the best interaction scores (ΔG, kcal/mol) for each snake venom defensin-K_v_1.3 channel docking were retained, and the interaction data was retrieved.

### Data analysis

Results were expressed as mean ± standard deviation. The experiments were analyzed using analysis of variance (ANOVA). The least significant difference test was performed when ANOVA showed significant differences (p < 0.05). All the graphs were plotted in Prism Graph Pad 9.

## Consent for publication

Not applicable

## CRediT authorship contribution statement

**David Melendez-Martinez:** Writing – review & editing, Writing – original draft, Visualization, Methodology, Investigation, Formal analysis, Conceptualization. **Adriana Morales-Martinez:** Writing – review & editing, Visualization, Methodology, Investigation, Formal analysis. **Iliana Vanessa Almanza-Campos:** Methodology, Investigation. **Francisco Sierra-Valdez:** Writing – review & editing, Resources, Funding acquisition, Formal analysis. **Miguel Borja:** Writing – review & editing, Resources, Methodology, Investigation, Funding acquisition. **Alejandro Carbajal-Saucedo:** Writing – review & editing, Supervision, Resources. **Christopher L. Parkinson:** Writing – review & editing. **Jorge Benavides:** Writing – review & editing, Supervision, Project administration, Funding acquisition, Conceptualization.

## Ethics approval and consent to participate

Not applicable.

## Funding

Jorge Benavides is supported by the Ciencia de Frontera 2023 grant (CF-2023-I-2019) from the Mexican National Council for Science and Technology (CONAHCYT). Miguel Borja is supported by the Ciencia de Frontera 2019 grant (FORDECYT-PRONACES/1715618/2020) from the Mexican National Council for Science and Technology. Francisco Sierra-Valdez acknowledges the financial support for this work by Federico Baur endowed chair in Nanotechnology (0020206BA1). Christopher L. Parkinson acknowledges funding from the U.S.A. National Science Foundation (DEB 1638879 & 1822417).

## Declaration of competing interest

The authors declare that they have no known competing financial interests or personal relationships that could have appeared to influence the work reported in this paper.

## Data Availability

Data will be made available on request.
