# Supplementary material for: Snake venom defensins: Defining the structural and functional characteristics of the toxin family
Source: J Struct Biol X. 2025 May 27;11:100129. doi: 10.1016/j.yjsbx.2025.100129 (PMC12162057; doi:10.1016/j.yjsbx.2025.100129)
Supplement: Supplementary Data 1 [file mmc1.docx]

**Supplementary figure 1. Structural stability of the *C. durissus* complex snake venom defensins.** Comparison of the Ctm_ducu_1 and Ctm_ducu_2 **A)** structure stability (RMSD) and **B)** C-α fluctuation (RMSF) in comparison to the *C. durissus* complex defensins. Both RMSD and RMSF were calculated from molecular dynamic simulations during 150 ns. The Ctm_ducu_1 and Ctm_ducu_2 are represented in blue and red, respectively. The mean ± standard deviation of the *C. durissus* complex defensins are represented by a black line and a grey shaded area, respectively.

**Supplementary figure 2. Structural stability of snake venom defensins. A)** Comparison of the snake venom defensins stability though RMSD, the stability was measured by molecular dynamic simulation during 150 ns. **B)** Structural flexibility was calculated through Cα fluctuation of the snake venom defensin residues. The sequences were aligned by MUSCLE algorithm.

**Supplementary figure 3**. **Effect of the mutations on the snake venom defensins stability.** The snake venom defensins stability was measured using the free energy change (ΔΔG, kJ/mol) as indicator, crotamine from *C. d. terrificus* (Ctm_dute_8) was used as a canonical structure to compare with the other snake venom defensin structures.

**Supplementary figure 4. Protein-protein molecular docking between the snake venom defensins and** **K_v_ 1.3 channel.** The heatmap illustrates the interaction relevance of each residue from snake venom defensin diversity when interacted with K_v_ 1.3 channel. It summarizes the number of interactions generated by residue of snake venom defensin in a white to red gradient. The sequences were aligned by MUSCLE algorithm. In the left margin represented the disulfide bridge framework (C4-C31, C11-37, C19-C38), and in the right margin identified the three basic-hydrophobic dyads (marked as D1, D2, and D3) described for crotamine: D1, Y1-K2; D2, R32-W33; D3, R34-W35.

**Supplementary figure 5. Interaction of the Snake venom defensins dyads with the K_v_ 1.3 channel pore residues.** The heatmaps illustrates the **A)** hydrogen bonds, **B)** hydrophobic interactions, **C)** electrostatic interactions, and **D)** saline bridges of each basic-hydrophobic dyad residues from snake venom defensin diversity when interacted with K_v_ 1.3 channel pore residues (G446-T454). It summarizes the number of interactions generated by dyad residue of snake venom defensin in a color gradient. The sequences were aligned by MUSCLE algorithm.
